# Oscillations and Boundaries in My Route Through the Hippocampal Cognitive Map

**DOI:** 10.1002/hipo.70052

**Published:** 2025-12-06

**Authors:** Neil Burgess

**Affiliations:** ^1^ UCL Institute of Cognitive Neuroscience University College London London UK; ^2^ UCL Queen Square Institute of Neurology University College London London UK

**Keywords:** boundary vector cell, grid cell, hippocampus, place cell, theta

## Abstract

I outline my personal journey in hippocampal research from joining the O'Keefe lab in 1991 to the present, with a focus on earlier experimental and computational investigations of spatial cognition and neural representations in rodents, with some reference to extensions to humans and to other aspects of cognition. These recollections are organized around place cells and the role of theta rhythmicity, the importance of environmental boundaries, the operation of a wider system for spatial memory and imagery, grid cells, and the neural mechanisms of navigation in humans. I conclude with some reflections on the collaborative and exciting ecosystem that supported all of these endeavors.

## 1 My Introduction to Hippocampal Research

My route towards the hippocampus started with Hopfield's (1982) model of associative memory. I was doing a PhD in theoretical physics, and the application of the statistical mechanics of “spin glasses” to memory rather than dilute magnetic alloys seemed too interesting to miss. I showed how some minor changes could give the Hopfield model more human‐like features of primacy and recency (Burgess et al. 1991), and my supervisor Michael Moore suggested I talk to someone who actually knew about memory, Graham Hitch who was also at Manchester University at that time. We produced a model of Baddeley and Hitch's (1974) phonological loop (Burgess and Hitch 1992), a component of working memory, but with links to long‐term memory (Burgess and Hitch 1999, 2006).

Following my PhD I took the opportunity of a 1‐year fellowship in Rome, to work with Stefano Patarnello at the IBM research centre there, attracted by the evolutionary algorithms he worked on (Patarnello and Carnevali 1989), ending up working on the use of constructive neural networks (i.e., networks which add additional neurons as part of the learning rule) for pattern recognition (Burgess 1994). While in Rome I decided to continue with neural network research, and that Sydney, Australia, would be a good next location. Of the faculty in Sydney that I had emailed, Bill (WG) Gibson was kind enough to reply, and he planned to apply for a grant on modeling the hippocampus. Aided by an introduction to the field from Alessandro Treves, who was visiting the CNR Instituto di Psicologia in Rome for a few days, I helped to write the grant application, which narrowly missed out on funding. Undeterred, I emailed the application to Bruce McNaughton, who I thought was in Boulder Colorado.

**FIGURE 1:**
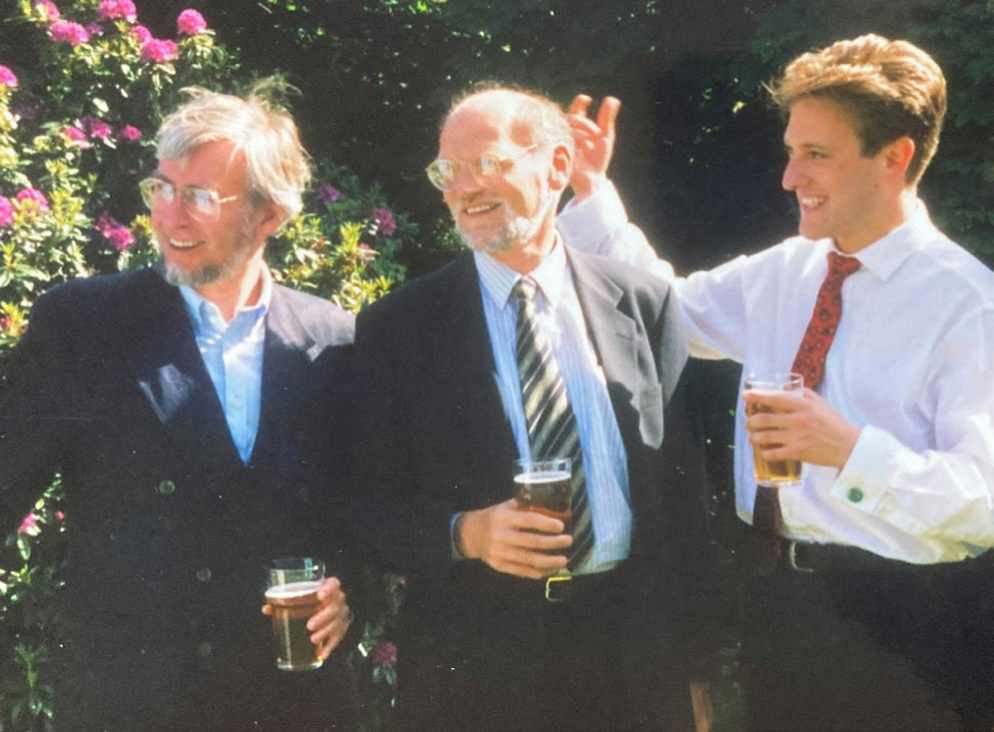
John O'Keefe, Graham Hitch and me at my wedding in 1997.

Bruce was kind enough to invite me to Tucson, where he had recently moved, and I spent a very interesting week with his lab, including Bill Skaggs and Matt Wilson, and colleagues Lynn Nadel and Carol Barnes. I planned to join them for a post‐doc but, back in Rome, I started going out with Cathy who was visiting from London, and decided to move there instead. I approached John O'Keefe at UCL, and was offered a post‐doc on a computational grant with John and Michael Recce, on the basis of an evening in a pub and a reference from Graham Hitch. On explaining my situation to Bruce, he emailed back “I reluctantly release you from your verbal contract” and I moved to London. I am pleased to say my decision was vindicated and Cathy and I are still married (Figure 1).

## 2 London and Theta Phase Precession

**FIGURE 2:**
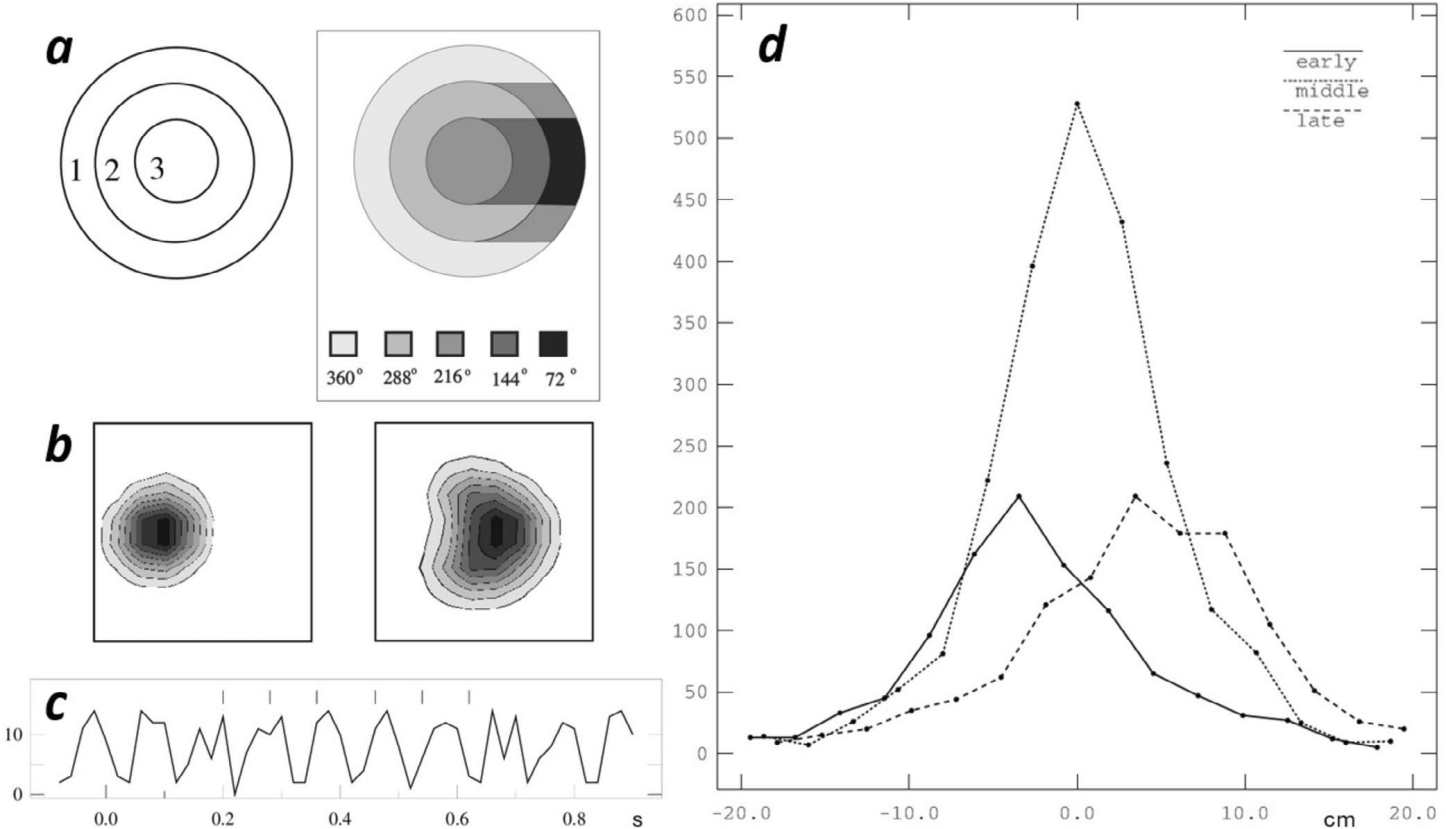
Population consequences of theta phase precession. (a–c) Simulation of a population of 484 place cells with evenly distributed place fields in a square environment, adapted from Burgess et al. (1992). (a) Each place cell fires a burst of 1, 2 or 3 spikes per 10 Hz theta cycle according to the rat's proximity to the centre of the field (left), at theta phases that get increasingly early as the field is traversed, illustrated for a trajectory from left to right (right; consistent with later experimental analyses, Jeewajee et al. 2014; Climer et al. 2013). (b) The population firing field (average of firing fields weighted by firing rates) moves from behind the rat (at the box center) at the early phase (left) to in front of it at the late phase (right). (c) The firing of each place cell (ticks, above) shows phase precession relative to the population firing rate (solid line, below). (d) Position of the place field center relative to the rat (at 0 on the *x* axis, moving from left to right) as a function of the theta phase of firing of the place cell (early, middle or late). Showing 4488 spikes from 13 place cells in three rats, adapted from Burgess et al. (1994).

When I arrived in London, John and Michael were working on their discovery of theta phase precession, whereby place cells fire at successively earlier phases of the local field potential (LFP) theta rhythm as the rat runs through the firing field (O'Keefe and Recce 1993). They proposed a “dual oscilIator” model in which the place cell has a higher intrinsic frequency than the LFP. I was interested in the consequences at the population level: that the place cells firing within each theta cycle would sweep forward from those with fields behind the animal to those with fields in front (Burgess et al. 1992). I verified this in experimental data (Burgess et al. 1994), followed by Bill Skaggs in a much larger dataset (Skaggs et al. 1996). A consequence of the waxing and waning of the firing within each field is that the higher intrinsic frequency of the place cells is consistent with the lower frequency of the overall population firing rate (which peaks at the middle phase when active place cells have place fields centred at the rat's location and fire at their peak rate; Burgess et al. 1992). This emphasizes both the independence of place cells' phase and rate codes, later verified in data recorded by John Huxter (Huxter et al. 2003), and that the “dual oscillators” are different aspects of the same system rather than independent frequencies, see also (Lengyel et al. 2003; Geisler et al. 2007) (Figure 2).

## 3 Boundaries and Remapping

After the initial demonstration of place cells (O'Keefe and Dostrovsky 1971; O'Keefe 1976), O'Keefe had worked to demonstrate their association to the concept of location, rather than simple perceptual responses. For example, with Andrew Speakman (who was a friendly presence in the lab when I arrived), he used cue rotation and cue removal manipulations to show that the place fields formed a coherent map that was oriented by the controlled cues but maintained after their removal and corresponded to the rats' choice of the goal arm in a plus maze (O'Keefe and Speakman 1987). However, the most systematic characterization of environmental influences on place cell firing came from Muller and Kubie (1987), although it was still unclear why a place cell fired in a given location. John was interested in the distinct effects of changing environmental size and shape, and combined both by recording from a set of four rectangular enclosures. Rather than the expected “remapping” of firing fields (see Kubie 2025), or responding at corresponding (scaled) locations across boxes, firing fields appeared to be superpositions of Gaussian responses tuned to distances to the surrounding boundaries (O'Keefe and Burgess 1996).

**FIGURE 3:**
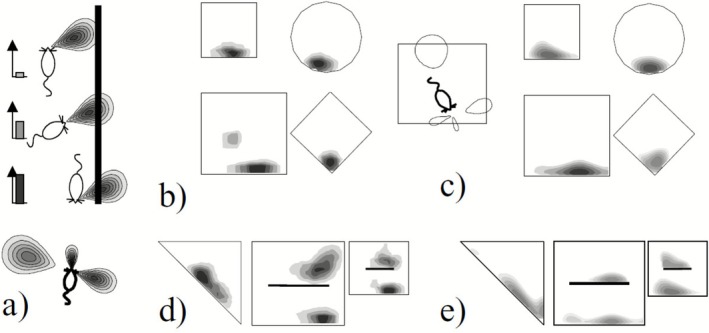
Boundary vector cells (BVCs) as inputs to place cells, adapted from Hartley et al. (2000). (a) BVCs are tuned to respond to environmental features at a specific distance and allocentric direction, with tighter tuning for shorter distances. (b) The firing of a place cell recorded in different shaped configurations of a morph box by Colin Lever can be modeled as the thresholded sum of 4 BVCs, as shown in (c). The model can predict the firing of this place cell in different configurations (e), with reasonable correspondence to the subsequent recordings in those configurations (d).

With my post‐doc Tom Hartley and Andrew Jackson (on a short rotation in the lab) we quantified how place fields could result from inputs from “boundary vector cells” (BVCs), each tuned to respond to the presence of a boundary at a specific distance and allocentric direction from the rat (Burgess et al. 2000; Hartley et al. 2000). This model could explain the distributions of shapes and sizes of place fields across different environments, assuming a bias towards shorter preferred distances. It also predicts a given cell's firing field after spatial re‐configurations of an environment, including the addition of an internal barrier, as recorded by Colin Lever (Hartley et al. 2000; Lever et al. 2002), or more complex manipulations (Grieves et al. 2018; Lee et al. 2025) (Figure 3).

Colin was a motivating force in this research; his enthusiasm and willingness to record from place cells before and after the addition of a barrier within the environment, was a source of great encouragement. He was interested in exploring these theoretical predictions further, and he went on to discover that the predicted BVCs actually existed, in recordings from the subiculum (Barry et al. 2006; Lever et al. 2009; see Lever 2025).

While I was visiting the Moser lab in 2008, Trygve Solstad was recording similar responses in the entorhinal cortex, although only shorter‐distance responses were apparent. Edvard Moser asked what the distinguishing feature of BVCs would be and I suggested inserting a barrier, and within a short time Trygve reported that the firing field showed the expected doubling (Solstad et al. 2008). A very exciting visit for me, although Edvard explained that he “would never name an experimental finding after a theoretical prediction, because the theory could be wrong, but the data are the data,” deciding on the term “border cell”. Recent quantification of BVC and border cell responses in the entorhinal cortex shows the border cells to be a subset of the BVCs (Muessig et al. 2024, fig. 6), and these cells have now been found in species from bats (Yartsev et al. 2011) to goldfish (Cohen et al. 2023).

The original plan had been to find representations that were different (‘remapped’) between two different shaped environments and to probe the intermediate shapes, for which I designed a flexible “morph box” whose shape and size could be varied. Colin found that place cells recorded in square and circular configurations initially showed similar firing fields, consistent with the BVC model (e.g., Figure 3b). However, as he continued to record over several days, place cells would slowly and independently remap between the two shapes—a learned distinction that lasted at least several months (Lever et al. 2002). Interestingly, recordings from intermediate (octagonal) shaped environments from animals trained in this way showed gradual transitions through intermediate shapes (unpublished observation, but see Leutgeb et al. 2005 for similar results). By contrast, exposure to initially different circular and square boxes followed by transfer to circular and square configurations of the morph box showed strong attractor dynamics when probed with intermediate shapes (Wills et al. 2005).

## 4 Egocentric‐Allocentric Translation, Memory and Imagination

With Sue Becker, who came to UCL for a sabbatical in 2000, we decided to model spatial memory in more detail. She already saw the hippocampus as a “generative” model, combining bottom‐up encoding of perception into memory and top‐down generation of imagery from memory (ideas later published in Becker 2005; see also Kali and Dayan, Káli and Dayan 2002). This view corresponded closely to my own ideas about the constructive nature of memory, in which memories are active reconstructions of what might have happened (Bartlett 1932), and its close links to imagery for events whether experienced or not.

We started by assuming that connections from vector cells (VCs, not being specific to boundaries) to place cell connections could be bi‐directional, so that place cells coding for a given location could reactivate VCs corresponding to the distances and allocentric directions of environmental objects. We added neurons coding for the visual appearances (texture) of different objects, and attractor dynamics to ensure that only one location could be represented at a time. This made for a medial‐temporal lobe that could pattern complete environmental information, but for both encoding and retrieval to make sense, the VCs needed connecting with representations from perception and to imagery, respectively.

**FIGURE 4:**
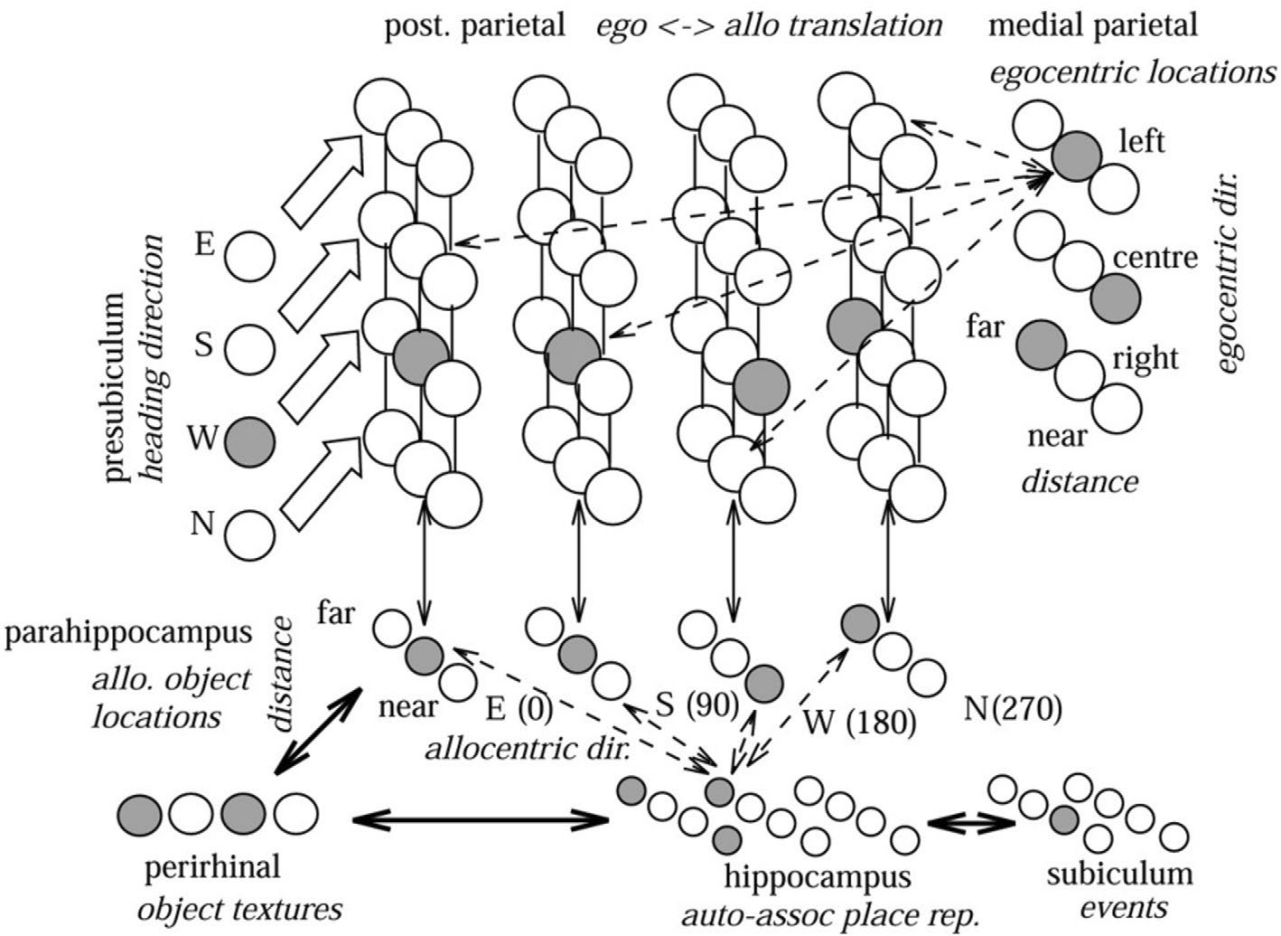
The model of spatial memory and imagery, adapted from Burgess, Becker et al. (2001). The model architecture. Note the allocentric encoding of direction (NSEW) in the parahippocampus, the egocentric encoding of directions (LR) in the medial parietal cortex, and the translation between them via head‐direction modulated neurons.

The remaining challenge for this model was that perception and imagery are egocentric, detailing what is left, right or ahead, whereas the firing of VCs corresponds to what is North, East, South or West (relative to head‐direction cells) irrespective of the orientation of the animal. Borrowing from Pouget and Sejnowski's (1997) model of gain field neurons for translation between frames of reference, we presumed that conjunctive (i.e., head‐direction modulated) VCs would translate between populations of ego‐centric and allo‐centric VCs according to head‐direction (e.g., if facing North then left = West) (Figure 4).

**FIGURE 5:**
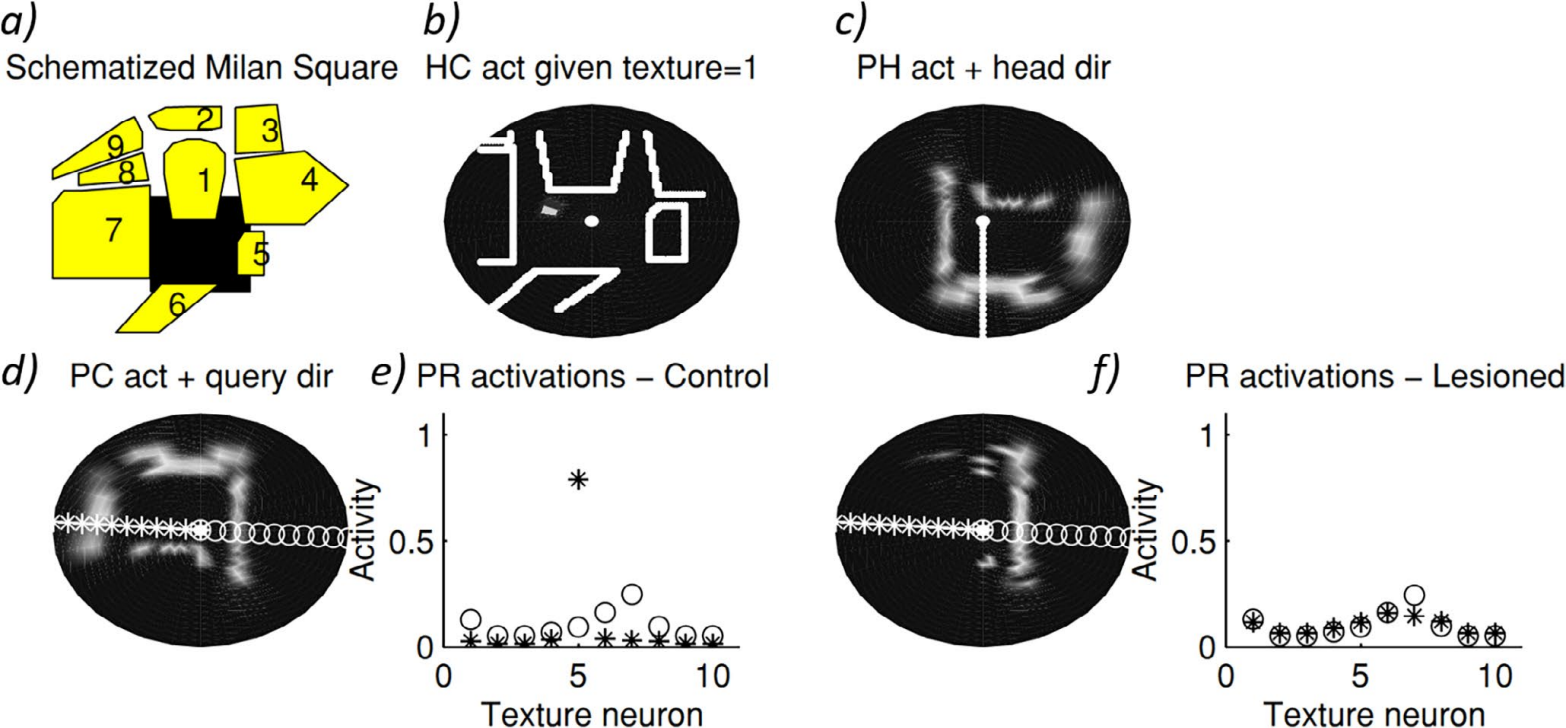
Simulation of retrieval of spatial information in the Milan square experiment of Bisiach and Luzzatti (1978), adapted from Becker and Burgess (2000). (a) Training consists of simulated exploration of the square (shaded area, North is up) allowing learning of connections between place cells (PCs), VCs and texture neurons. Recall and imagination: The system is cued to imagine being near the Cathedral (i.e., the perirhinal cell for the building 1 texture, and VCs for a building at a short distance North are activated) and the system settles. (b) The hippocampus settles to a location in the Northwest corner of the square (hippocampal cell activity is shown as the brightness of the pixel corresponding to the location of each cell's place field; a central origin and building outlines have also been added). (c) The parahippocampal activity, VCs correctly retrieve the locations of the other buildings (firing is shown as the brightness of the pixel for the location encoded by each cell, relative to the subject at the center). A line indicates that the imagined head direction is South. (d) Medial parietal cell activity: The parahippocampal map has been correctly rotated given head direction South (straight ahead is up), stars indicate a query direction of attention to the left, circles to the right. (e) Perirhinal cell activations correctly showing building 5 to the left (stars) and building 7 to the right (circles). (f) Effect of a right parietal lesion on the medial parietal representation (left: Note lack of activation on the left) and PR (right: Note decrease in activation of building 5 when inspection is to the left, stars).

The resulting model (Becker and Burgess 2000; Burgess, Becker, et al. 2001) can use the long‐term knowledge of the topographical layout of a familiar environment to generate imagery from a viewpoint (place cells) and viewing direction (head direction cells). Lateralised damage to the translation or egocentric networks would produce hemispatial representational neglect, as in the famous Milan Square experiment (i.e., inability to imagine the left side of the square following right hemisphere damage, often including posterior parietal cortex; Bisiach and Luzzatti 1978) (Figure 5). Later extensions of this model, with Patrick Byrne and Andrej Bicanski, cover many other aspects of spatial memory and imagery, and identify the ego–allo translation with retrosplenial cortex, but retain the basic mechanism (Byrne et al. 2007; Bicanski and Burgess 2018).

This model posited several distinct cell types: egocentric and allocentric “vector” cells responding to boundaries and objects, with their head‐direction gain‐modulated versions for translation, and “vector trace” responses to a removed object (Poulter et al. 2021) due to pattern completion. The majority has now been discovered, including egocentric BVCs (Hinman et al. 2019; Alexander et al. 2020; Gofman et al. 2019), head‐direction modulated BVCs (Peyrache et al. 2017; Gofman et al. 2019), object vector cells (Høydal et al. 2019) and egocentric object vector cells (Wang et al. 2018), reviewed by Bicanski and Burgess (2020). In addition, the diagonal connectivity between egocentric direction neurons and gain‐field neurons arranged by allocentric direction (Figure 4, dashed arrows to top right; Bicanski and Burgess 2018, fig. 2S1) has been identified in the circuit translating egocentric to allocentric movement direction in the fly (Lu et al. 2022, fig. 3g).

The role of the hippocampus in generating imagery has also become mainstream, not least through the work of Demis Hassabis during his PhD with Eleanor Maguire, showing that hippocampal amnesia impairs the ability to imagine coherent spatial scenes (Hassabis et al. 2007). Demis had shown a keen interest in the model, and later (with many others, including Caswell Barry) showed that deep recurrent networks could learn a similar mechanism when simply trained to predict the video input to a moving agent (Uria et al. 2020). Generative models are now extensively used to explain phenomena such as consolidation, semanticisation and replay (Káli and Dayan 2002; Pezzulo et al. 2017; Van de Ven et al. 2020; Fayyaz et al. 2022; Spens and Burgess 2024; Dong et al. 2025), as well as the formation of spatial firing patterns including grid cells (Whittington et al. 2020).

## 5 Grid Cells

Prior to their seminal discovery of grid cells, I had met Edvard and May‐Britt Moser when they came to visit the O'Keefe lab in the mid/late 1990s, learning tetrode recording with John and Colin, and Jim Donnett's Axona recording system. I remember May‐Britt's cry of “where is Neil, I will kiill him” when she thought my tetrode interface software (Tint) might have deleted her first recording. As noted by Kate Jeffery (Jeffery 2025), we can all remember reading about grid cells in the Hafting et al. (2005) paper, and the excited discussions they provoked. In retrospect, not only had Kate recorded grid cells hidden amongst other data, as it seems likely that some of Francesca Cacucci's theta‐modulated place‐by‐direction cells in pre‐ and para‐subiculum (Cacucci et al. 2004) were conjunctive grid cells. However, without Menno Witter to direct us to the locations where responses have the finest spatial scales, or Bill Skaggs to suggest recording in larger boxes, the periodic structure of these firing patterns remained obscure to us. This discovery, proof of internally generated structure within the brain, provided a massive boost of intellectual energy to the whole field.

**FIGURE 6:**
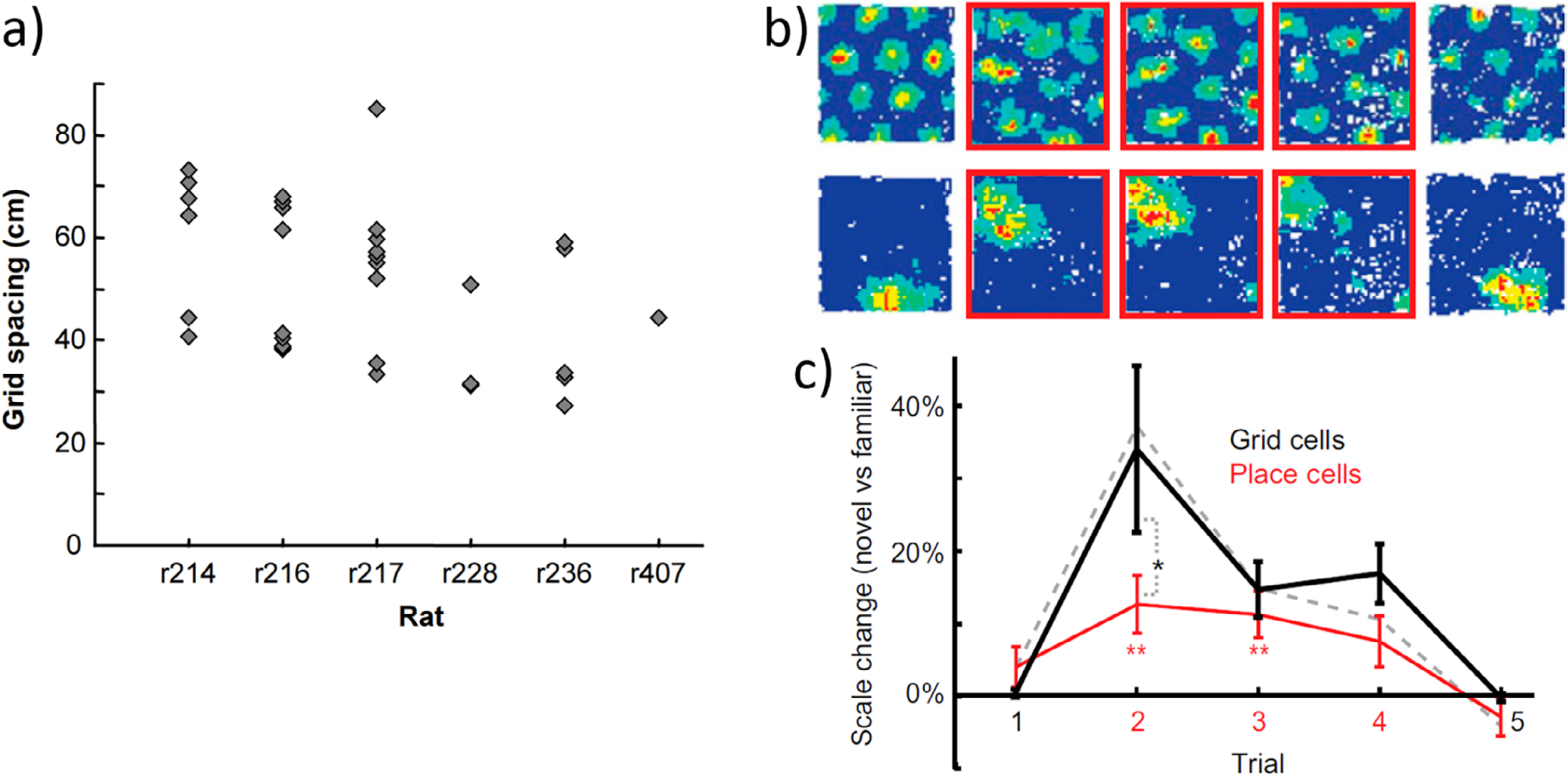
Grid cells. (a) Evidence for modules. Adapted from Barry et al. (2007, fig. 3 which mistakenly shows grid scale in units of 2 cm). (b) Novelty expansion of grid cell (above) and place cell (below) firing patterns across successive trials in a familiar and novel box (red outline), with change in scale shown below for all cells (c), adapted from Barry et al. (2012).

Caswell Barry, just starting his PhD with Kate and me, was keen to record grid cells in a replication of the stretchy box experiment (O'Keefe and Burgess 1996), to test the apparently rigid crystalline firing patterns in a situation when place cell responses were flexible. With Robin Hayman, he found that the grid patterns also stretched, although not quite as much as the place cells had, and that the effect was specific to stretching a familiar box, rather than using a new one of a different size (Barry et al. 2007). He also noticed that grid scale was quantised within each animal—the first evidence that they form discrete modules; see Figure 6.

Caswell, now as a post‐doc in my lab, followed up the experience dependence seen in this experiment, showing that grids have much larger scales when the rat is put in a new environment, coinciding with place cell remapping, and that the grid scale shrinks with experience (Barry et al. 2012, fig. 6). Grid cells were thought to mediate path integration (McNaughton et al. 2006), whereas place cell firing patterns are very similar across boxes of similar appearance and orientation, as per the BVC model, *even if the rat can walk between them* (Skaggs and McNaughton 1998; Spiers et al. 2015). Again, Caswell sought to test this apparent contradiction, with Francis Carpenter who became Caswell's first PhD student as Caswell set up his own lab. He found that, like place cells, grid patterns in two identical boxes were initially copies of each other, but over many days of walking between the boxes, slowly adjusted towards becoming a single global grid across both boxes (Carpenter et al. 2015). This adjustment happens over a similar timescale to that over which place cells slowly remap between similar environments (Lever et al. 2002).

The rescaling experiments suggest that grids attempt to maintain both their own natural scale and associations between place and grid cells that form with experience of a new environment. This is consistent with the idea that grid and place cells combine self‐motion and environmental inputs to estimate location (O'Keefe and Burgess 2005). Although coupled, the grid cells should reflect self‐motion while place cells reflect environmental inputs, which is hard to test as both types of input usually change together. With Francesca Cacucci, John King, Yi Lu and Guifen Chen, we developed a virtual reality system in which mice can rotate as well as translate, following (Holscher et al. 2005; Aronov and Tank 2014), allowing the recording of directional or grid‐like firing patterns (Chen et al. 2018). Simultaneously recording place and grid cells while changing the gain at which visual inputs move in response to physical self‐motion shows that indeed, the place cells are more influenced by (visual) environmental inputs and the grid cells by physical self‐motion inputs (Chen et al. 2019), although both cell types show influences of both inputs (Chen et al. 2013). Overall, these cells resemble components of a system for Simultaneous Localisation and Mapping, as explored by Talfan Evans (Evans and Burgess 2019), see also (Ocko et al. 2018; Milford and Wyeth 2008).

## 6 Back to Theta: Oscillatory Interference and Theta Sweeps

Two puzzles had been bothering me on and off for several years prior to the discovery of grid cells. One of these was the absence of evidence for the movement signal required for continuous attractor models of place cells and path integration (Zhang 1996; Conklin and Eliasmith 2005). Although cells with firing rate modulation by head direction (e.g., Taube 2025) and by running speed (e.g., McNaughton et al. 1983) were known, neither encodes the movement velocity required for path integration, as later demonstrated by Mike Hasselmo's lab (Raudies et al. 2015). The other was why the “dual oscillator” model (O'Keefe and Recce 1993) should produce unimodal place cell firing, when a multi‐peaked interference pattern would be expected.

The discovery of grid cells immediately reminded John and me of interference patterns (O'Keefe and Burgess 2005), see also (Blair et al. 2007). The dual oscillator model requires an intrinsic frequency that increases from the population (or “baseline”) frequency, which is a natural consequence of theta phase precession (see Section 2). For place cells, firing phase correlates with distance traveled through the firing field (O'Keefe and Recce 1993), indicating that the difference between intrinsic and population frequency increases with running speed. With Caswell, aided by discussions with Kate and John, we realized that a frequency difference that was proportional to the component of velocity in a specific “preferred direction” would produce periodic bands (i.e., plane waves or Fourier components) as a phase code, resulting in bands of firing if summed with the population oscillation. Summing such “velocity controlled oscillators” with preferred directions at multiples of 60° would produce grids, whose scale reflects the constant of proportionality. The model seemed elegant but somewhat far‐fetched and we presented it as a poster for the Computational Cognitive Neuroscience Conference (Burgess et al. 2005).

Mike Hasselmo attended CCNC and was excited by the model's explicit prediction of a link between intrinsic oscillations and grid scale (Hasselmo 2013, 2025). A year or so later he encouraged me to write up the full model (Burgess et al. 2007; Burgess 2008). He needed something to cite in some work with Lisa Giocomo, showing that intrinsic frequencies in mEC slices reflect their dorsoventral location, corresponding to the dorsoventral gradient in grid scale (Giocomo et al. 2007). The enthusiasm of Mike and Tad Blair was a real motivation to continue to develop the “oscillatory interference” model, and with Dan Bush we showed how oscillatory interference could indeed drive path integration of the activity bump within continuous attractor models of grid cells, with theta phase precession (Bush and Burgess 2014).

“All models are wrong, but some are useful” (Box 1976), and the simplicity of the oscillatory interference model certainly facilitates predictions (Burgess 2008), although the *difference* between intrinsic and population frequency can be hard to measure. Ali Jeewajee showed that intrinsic and population or local field potential frequencies increase with speed and decrease with grid scale appropriately (Jeewajee et al. 2008). Tad Blair's group showed that “theta cells” might show the predicted intrinsic frequency tuning to speed and direction (Welday et al. 2011), while Colin Lever and colleagues showed changes in the theta frequency–speed relationship consistent with the effect of novelty on grid scale (Wells et al. 2013). With Julija Krupic, we saw signs that the spatially modulated firing patterns of neurons in mEC and adjacent parasubiculum are composed of periodic bands, only the most regular of which qualify as grid cells (Krupic et al. 2012).

More recent theoretical accounts of grid cell firing patterns suggest that they act as eigenvectors of the matrix of transitions between states: providing a basis set on which prediction of future state occupancy corresponds to a simple re‐weighting (Corneil and Gerstner 2015; Stachenfeld et al. 2017; Baram et al. 2018; Whittington et al. 2020). In this context, with Changmin Yu and Tim Behrens, we showed how using Fourier components (periodic bands of firing rate or firing phase) to encode location could allow prediction of the effects of movements in different directions (i.e., path integration; Yu et al. 2020).

We still do not know the origin of the movement signal that is integrated in path integration, albeit that oscillatory interference provides one potential solution. A related solution is suggested by “theta sweeps,” that is, the firing of place cells within each theta cycle sweeping from those with firing fields behind the animal to those with fields in front of it (see Section 2), with potential application to humans (Bush et al. 2017; Bush and Burgess 2020).

By recording large numbers of place cells, Johnson and Redish (2007), could decode the location of the rat within short timescales, decoding forward trajectories which sampled the possible directions on the maze at choice points. Importantly, studies in rats moving backwards show that theta sweeps describe trajectories in the direction of movement rather than head direction (Maurer et al. 2014; Cei et al. 2014). Theta phase coding has now been recorded in humans (Qasim et al. 2021) and bats (Eliav et al. 2018), with the latter also showing theta sweeps during flight (Forli et al. 2025).

Thus, theta sweeps appear to reflect more than just current or potential movements, often alternating across left and right options in a maze (Johnson and Redish 2007; Kay et al. 2020). Accordingly, theta‐sweep‐like mechanisms have appeared in various models of exploratory or searching behavior (Erdem and Hasselmo 2012; Kubie and Fenton 2012; Bush et al. 2015).

Very recently, Vollan et al. (2025) showed that theta sweeps alternate about the forward direction during foraging in open fields, and are driven by a circuit of theta‐modulated directional cells projecting to grid cells via conjunctive directional grid cells, and thence to place cells. Zilong Ji showed that alternating theta sweeps within this network can be simulated by assuming continuous attractor dynamics in the directional and grid cells, together with theta modulation and firing rate adaptation (Ji, Chu, et al. 2025b). This model builds on earlier work with Tianhao Chu and Si Wu (Chu et al. 2024), avoiding the coding of self‐motion by using the change in sensory input to move the activity bump, following Tsodyks et al. (1996). One prediction of this model is that theta‐modulated directional cells should show phase precession when turning through their preferred direction, which we confirmed in data recorded by Kate, Eleanora Lomi and Anna Mitchell from anteroventral thalamus (Ji, Lomi, et al. 2025a). The alternating theta sweeps also provide an explanation for the “theta‐cycle skipping” observed in mEC (Brandon et al. 2013; Jeffery 2025) and anteroventral thalamus (Ji, Lomi, et al. 2025a).

During goal‐directed navigation, it seems that theta sweeps may answer one of the longest‐standing puzzles in hippocampal navigation, that is, beyond encoding the animal's own location, orientation and movement, how is the goal encoded? Theta sweeps are longer when the goal location is further away (Wikenheiser and Redish 2015) and are better learned (Zheng et al. 2021), however potential movement directions and goal direction are hard to separate on narrow‐armed mazes, while animals in open fields will both orient and move towards the goal. By using data recorded by Jake Ormond and John from the “honeycomb maze” which dissociates movement, head and goal directions (Ormond and O'Keefe 2022), Changmin was able to unambiguously demonstrate that theta sweeps indicate the goal direction rather than head or movement directions, which Zilong showed to be consistent with the presence of a top‐down goal‐direction input to the theta‐modulated directional cells (Yu et al. 2025).

## 7 Spatial Memory and Virtual Reality in Humans

Throughout my work on rodent hippocampus (the focus here), I was also keen to translate the findings to humans, where possible. The arrival of the Functional Imaging Lab (FIL) at UCL Queen Square, opposite the National Hospital of Neurology and Neurosurgery, and the establishment of the Institute of Cognitive Neuroscience next door (where I worked), provided an exciting new opportunity to study navigation in head‐fixed volunteers in brain scanners and in neurological patients. Inspired by Aguirre et al. (1996) use of video games, I modified Duke Nukem to make a small virtual town within which to test navigation and episodic memory, aided by Jim Donnett's machine code skills to extract behavioral variables from the game.

Eleanor Maguire, who had recently arrived at the FIL, ran a Positron Emission Tomography experiment on participants navigating through the town, showing that right hippocampal activity was the best correlate of navigational accuracy, followed by right posterior parietal cortex (Maguire et al. 1998). For his PhD, Hugo Spiers tested temporal lobectomy patients, showing deficits in navigation in right temporal lobectomy and in remembering contextual aspects of events experienced in the town in left temporal lobectomy (Spiers, Burgess, Maguire, et al. 2001), while a bilateral developmental amnesic, Jon, tested with Faraneh Vargha‐Khadem, was impaired in both types of tests (Spiers, Burgess, Hartley, et al. 2001). John King later showed that Jon was specifically impaired in object location memory when tested from a shifted viewpoint, but could recognize the correctly located object from the same viewpoint as presentation (King et al. 2002). Once fMRI scanners arrived at the FIL, we showed that remembering the spatial context of virtual events corresponded to activity in the areas implicated by the Becker and Burgess model (Burgess, Maguire, et al. 2001).

Another review would be required to cover all of the related work on spatial cognition using virtual reality with fMRI or intracranial recording that followed from this time, and from Russel Epstein and Nancy Kanwisher's (Epstein and Kanwisher 1998) paper on the parahippocampal “place area,” including Veronique Bohbot, Nora Newcombe, Jack Loomis, Alain Berthoz, Timothy McNamara, Mike Kahana, Chantal Stern, Liz Chrastil, Bill Warren, Mary Hegarty, Thomas Wolbers, Sang‐Ah Lee, Jan Wiener, Josh Jacobs, Weimin Mou, Nanthia Suthana, Nicolai Axmacher, Liang Wang and many more (apologies for not covering this field properly here).

Around this time, there was a strong position in the literature that spatial cognition reflected purely egocentric representations (i.e., perceptual representations, and spatial updating via egocentric self‐motion signals; Wang and Spelke 2002). To address this, we extended Wang and Simons' (1999) paradigm to show that both of these processes co‐exist with (allocentric) representations of object locations relative to environmental cues in supporting behavior (Burgess et al. 2004). Elizabeth Spelke very graciously let me know that this and other experiments reviewed in (Burgess 2006) had changed her mind on this topic.

Tom Hartley was keen to examine predictions of the BVC model for human memory, so we made the assumption that memory for spatial location reflects place cell firing (i.e., you return to a location by matching the current pattern of place cell firing to the pattern at that location). We asked people to return to a location in a virtual arena (created by Tom using Quake2) after changing the shape of the arena. The BVC–place cell model best captured the distribution of responses, for example, better than matching views, or distances to features such as corners (Hartley et al. 2004), see related work by (Keinath et al. 2021). He also showed that performance at navigating a virtual town via a well‐learned route versus shortest paths correlated with caudate versus hippocampal activity (Hartley et al. 2003).

Christian Doeller continued this line of work, with John King who had designed a virtual object‐location task within a circular arena (with distant landmarks for orientation), investigating the learning of object‐locations relative to the environment boundary versus relative to a discrete landmark correlated with activity in the hippocampus versus striatum (Doeller et al. 2008) and used an incidental learning rule versus a reinforcement learning rule (Doeller and Burgess 2008). I am indebted to Peter Dayan and Mortimer Mishkin for selflessly offering lengthy advice on these two papers. The relative contributions of striatal and hippocampal learning in these experiments were captured in a computational model by Jesse Geerts (Geerts et al. 2020).

With the discovery of grid cells, Christian and Caswell were keen to find some way of detecting them in humans. Caswell noticed that the orientations of grids in different modules (see Figure 6a) were significantly aligned (albeit less tightly than within modules; Stensola et al. 2012), so I suggested a quadrature filter approach to finding six‐fold modulation of fMRI activity by running direction during virtual navigation. Christian found that indeed there was such modulation, not only in entorhinal cortex, but throughout the autobiographical memory system (Doeller et al. 2010). We assumed that the different patterns of grid cell firing on versus off axis would affect the fMRI signal given its highly non‐linear relation to neural activity (we now think that firing rate adaptation causes a difference in firing when running on versus off axis, observable in grid cells in rats, a work in progress). We interpreted the addition of grid cells to the BB model as allowing shifts in the viewpoint established by place cells (Bicanski and Burgess 2018), with Aidan Horner showing that directions of imaged movement also produce the grid‐like fMRI modulation (Horner et al. 2016).

## 8 Reflections

The last 50 years of research into the extended hippocampal system has provided a dazzling breakthrough into the neural mechanisms of spatial cognition. It has been a privilege to have seen the last 35 years via involvement with several of the relevant people and discoveries. A particularly exciting and collaborative ecosystem developed at UCL out of the O'Keefe lab, flourishing from the late 90s and through the noughties. This period featured games of softball in Regent's Park, dinner parties at Eileen and John's flat with semi‐industrial production of Margheritas, and the progression of several labs to independence within UCL while maintaining a powerful and friendly pooling of resources and know‐how. This environment was further enhanced by plentiful American visitors such as Lynn Nadel, Gyuri Buzsaki, Bob Muller, John Kubie, Jim Knierim, Andre Fenton, while the Mosers initiated a European consortium “SpaceBrain” involving regular meetings with many researchers in Europe including Joszef Csicsvari, Alessandro Treves, Hannah Monyer, Menno Witter, Misha Tsodyks and Nachum Ulanovsky.

For me, it has been an amazing ride through cognitive and computational neuroscience. I have been particularly fortunate in the way this field has facilitated interaction between theory and experiment, replete with highly interpretable neural representations and relatively straightforward links to cognition and behavior. Importantly, there are also good prospects for translating some of the fundamental insights gained towards understanding various neurological and psychiatric conditions. For example, with Chris Brewin, we have updated the “dual representation” account of posttraumatic stress disorder (Jacobs and Nadel 1998; Brewin 2001), see (Brewin et al. 2010), with several predictions tested by James Bisby (e.g., Bisby and Burgess 2017). Dan Bush and Rick Adams found that medial temporal–prefrontal theta phase coupling (Adams et al. 2020) and, with Laura Convertino, entorhinal grid‐like representation (Convertino et al. 2023) are reduced in Schizophrenia. Finally, in work with Dennis Chan, Chris Bird and Andrea Castegnaro, our hippocampal‐dependent tests of spatial memory (Hartley et al. 2007) and path integration are proving highly sensitive to Alzheimer's disease (Bird et al. 2010; Howett et al. 2019; Castegnaro et al. 2023).

## Funding

This work was supported by Wellcome Trust, 202805/Z/16/Z.

## Data Availability

Data sharing not applicable to this article as no datasets were generated or analysed during the current study.
